# Continuous Intravenous Flumazenil Infusion Used in Iatrogenic Chlordiazepoxide Overdose in the Setting of Alcoholic Withdrawal Syndrome Management

**DOI:** 10.7759/cureus.10648

**Published:** 2020-09-25

**Authors:** Giselle S Volney, Robyn Scatena

**Affiliations:** 1 Internal Medicine, Norwalk Hospital, Norwalk, USA; 2 Pulmonary and Critical Care, Norwalk Hospital, Norwalk, USA

**Keywords:** chlordiazepoxide, benzodiazepine, overdose, flumazenil, infusion, alcohol withdrawal syndrome

## Abstract

Chlordiazepoxide is a benzodiazepine that is widely used in the treatment of alcohol withdrawal syndrome (AWS). Flumazenil is a competitive antagonist at the benzodiazepine receptor site and is the drug of choice for the treatment of benzodiazepine overdose. Reversal of benzodiazepine overdose is usually achieved by the use of a flumazenil bolus; however, the continuous infusion has been used when concomitant medical conditions may lead to delayed metabolism of the benzodiazepine involved. We present a patient with AWS, with inadvertent chlordiazepoxide overdose treated effectively with a prolonged flumazenil infusion.

## Introduction

Alcohol withdrawal syndrome (AWS) is a central nervous system hyperexcitability state, which can lead to significant morbidity and mortality [1]. AWS is common in patients with alcohol use disorder after periods of decreased alcohol intake or cessation. Benzodiazepines, including chlordiazepoxide, a long-acting benzodiazepine, are the first-line drug class used in alcohol withdrawal syndrome [1]. A symptom-triggered approach in conjunction with scoring systems, such as the Clinical Institute Withdrawal Assessment (CIWA) of Alcohol Scale, Richmond Agitation-Sedation Scale (RASS), or Riker score, has been adopted in clinical practice; the next dose of benzodiazepine is administered when the patient starts to display recrudescent AWS with the goal to reduce the total dose of benzodiazepine required and the length of hospital stay [2]. With a symptom-triggered approach, chlordiazepoxide can be given in 50 mg to 100 mg increments, as triggered by symptoms for a maximum of 300 mg per day [3]. Chlordiazepoxide is metabolized in the liver, and patients with chronic liver disease may experience elevated levels of chlordiazepoxide and its active metabolites for extended periods of time after initial use [4]. Obesity and acute kidney injury can also result in delayed elimination and thus elevated levels of benzodiazepines for prolonged periods of time [5], which can contribute to benzodiazepine overdose. Prolonged sedation and respiratory insufficiency are symptoms of chlordiazepoxide overdose [6]. Flumazenil is a benzodiazepine receptor antagonist and is first-line for the treatment of benzodiazepine overdose [4]. Flumazenil, administered as a continuous infusion, has been used effectively in clinical practice for the management of severe benzodiazepine overdose with minimal side effects [4,7].

## Case presentation

A 63-year-old African American female patient with a past medical history significant for alcohol use disorder, multiple hospital admissions for alcohol intoxication and alcohol withdrawal syndrome, obesity, untreated hepatitis C, and chronic obstructive pulmonary disease presented to our hospital with alcohol intoxication with a blood ethanol level of 279 mg/dL and bilateral pneumonia. On the third day of hospitalization, a chlordiazepoxide-based alcohol withdrawal protocol using a combination of fixed and symptom triggered dosing was initiated for control of AWS symptoms. The symptoms of AWS worsened, and the patient began to experience disorientation, auditory and visual hallucinations, and worsening hypertension despite chlordiazepoxide administration leading to rapidly escalating doses. Over a four-day span, 1375 mg of chlordiazepoxide was administered for symptom management. The patient then became lethargic with little arousal to physical stimuli. Acute encephalopathy did not improve with the cessation of chlordiazepoxide. Arterial blood gas showed a pH of 7.38 and a partial pressure of carbon dioxide of 53.5 mmHg. Electroencephalography, computer tomography of the brain without contrast, magnetic resonance imaging (MRI) of the brain without contrast, and lumbar puncture revealed no explanation for the patient's acute encephalopathy. Computed tomography of the abdomen showed an enlarged liver measuring approximately 24 cm in transverse diameter and moderate hepatic steatosis. Blood, urine, sputum, and cerebrospinal fluid gram stain and culture were negative. The patient was treated with high-dose IV thiamine with no improvement in mental status. Richmond Agitation Sedation Scale (RASS) score was -4 without sedation.

The patient was admitted to the intensive care unit (ICU) for acute encephalopathy and respiratory depression requiring mechanical ventilation. Vital signs on arrival to the intensive care unit (ICU) included a blood pressure of 135/82, pulse of 92, respiratory rate of 22, and oxygen saturation of 97%. Examination showed a lethargic obese African American female not responding to commands or verbal stimuli but withdrawing to pain. The examination of the chest showed bibasilar crackles on auscultation. The body mass index (BMI) was 35.4. Complete blood count upon arrival to the ICU included macrocytic anemia with a hemoglobin of 11.7 g/dl and mean corpuscular volume (MCV) 117.9 fL as well as a platelet count of 114,000/μL. Albumin 2.5 g/dL, total bilirubin 0.8 mg/dL, aspartate aminotransferase (AST) 34 unit/L, and alanine aminotransferase (ALT) 13 unit/L. Ammonia was 41 umol/L. The international normalized ratio (INR) was 1.24. Creatinine was 0.62 mg/dL. Arterial blood gas showed a pH of 7.23, partial pressure of carbon dioxide of 72.0 mmHg, and partial pressure of oxygen of 63 mmHg. There were no significant laboratory changes when compared to the presentation (Table 1).

**Table 1:** Pertinent laboratory on admission to the medical floors to admission to the intensive care unit

	On admission to medical floors	Admission to the intensive care unit
Total bilirubin mg/dL	1.2	0.8
Aspartate aminotransferase until/L	30	34
Alanine aminotransferase unit/L	36	13
Creatinine mg/dL	0.57	0.62

A trial of flumazenil 0.25 mg intravenously was given as a single dose with an instantaneous improvement of mental status; the patient was awake, responsive to questions, and able to follow commands. The effect wore off after a few minutes and the patient returned to a RASS of -4. The second trial of flumazenil was attempted with a similar response. The patient was diagnosed with chlordiazepoxide-induced encephalopathy and started on a flumazenil infusion, which was up-titrated from 0.25 mg/hour to 0.6 mg/hour with significant improvement in neurological status. The patient was able to be extubated and transitioned to noninvasive mechanical ventilation and then to a nasal cannula. After three days of flumazenil 0.6 mg/hour, attempts were made to titrate off flumazenil continuous infusion at a rate decrease of 0.1 mg/day, however, at 0.3 mg/hour, there was a deterioration in the patient's mental status with worsening lethargy. Flumazenil 0.5 mg/hour was resumed. The patient remained on the flumazenil continuous infusion for a total of 14 days and was successfully titrated off. There were no apparent adverse effects of the flumazenil continuous infusion. Blood chlordiazepoxide and nordiazepam levels 21 days post final administration of chlordiazepoxide was 513 ng/mL (therapeutic reference range 500-3000 ng/mL) and 324 ng/mL (therapeutic reference range 100-1500 ng/mL). Repeat urine benzodiazepine testing was positive 35 and 55 days after the last administration of chlordiazepoxide. The patient was transferred from the ICU after 27 days and discharged from the hospital 60 days post the last administration of chlordiazepoxide with no adverse effects from flumazenil.

## Discussion

Alcohol withdrawal syndrome (AWS) is an umbrella term for the signs and symptoms that patients with alcohol use disorder may experience upon cessation or reduction of alcohol intake [2]. Symptoms of AWS include hallucinations, seizures, and delirium tremens. Medications with gamma-aminobutyric acid (GABA) receptor activity, such as benzodiazepines, are first-line therapy for AWS [1]. 

Patient responsiveness to doses of benzodiazepines varies widely. Some patients display a relative resistance to benzodiazepines, often leading to escalating doses of drug administration [2]. At the dose required to control AWS symptoms, benzodiazepines can produce sedation, delirium [2], and acute encephalopathy [4]. Obese patients are at increased risk for these adverse effects, given the increased volume of distribution and marked distribution into adipose tissue, which prolongs the elimination of benzodiazepines [8]. Patients with chronic liver disease are further at risk for the prolonged excretion of benzodiazepines, likely secondary to impaired hepatic metabolism and the persistence of active metabolites, as well as increased free drug concentration due to the hypoalbuminemic states [9]. 

The choice of benzodiazepine should be determined based on the patient's concomitant medical conditions and the metabolism characteristics of individual benzodiazepines [6].* *Long-acting benzodiazepines, such as chlordiazepoxide and diazepam, and short-acting benzodiazepines like lorazepam and oxazepam are the most studied benzodiazepines used in AWS [10]. Longer half-life benzodiazepines may be chosen, as they often provide a smooth course of treatment with a lower risk of rebound symptoms, which occur late during withdrawal [10]. Chlordiazepoxide, with a half-life of 24 to 48 hours, is one of the most frequently used benzodiazepines for alcohol withdrawal. Chlordiazepoxide has a long duration of action and converts to active metabolites in the liver. These metabolites can accumulate and complicate treatment with increased risk of hepatic encephalopathy [6]. Desmethyldiazepam, a minor metabolite of chlordiazepoxide, has a half-life of as long as 200 hours [4]. Acute encephalopathy with decreased arousal requiring invasive mechanical ventilation has been described in patients with chronic alcoholism and alcoholic liver disease treated for AWS with high doses of chlordiazepoxide [4]. Shorter-acting benzodiazepines like lorazepam have no active metabolites and are less affected by the hepatic microsomal pathways, as they are metabolized by conjugation and do not undergo biotransformation in the liver. Lorazepam has been shown to be non-inferior to chlordiazepoxide in the management of AWS and is generally preferred in the treatment of AWS in patients with alcoholic liver disease [11].

Improvement in mental status after flumazenil injection is diagnostic for benzodiazepine overdose. A single dose of flumazenil 1 mg intravenously can show significant improvement in arousal, however, the effect may be short-lived, with subsequent deterioration of mental status in patients with severe overdose [4]. Flumazenil has a short elimination half-life and a short duration of activity, as it undergoes rapid hepatic metabolism and elimination [12]. The clearance of flumazenil, however, has shown a 25%-60% reduction in patients with moderate to severe liver dysfunction [4]. The reversible effect on mental status acts in a dose-dependent manner [12]. When repeated single doses of flumazenil fail to maintain alertness, a continuous infusion titratable to mental status can be used [4]. In patients with severe benzodiazepine overdose, who presented with coma and who were aroused by a single injection of flumazenil 1 mg intravenously, an infusion of 0.5 mg of flumazenil per hour was shown to prevent relapse into the coma without significant adverse effects [7]. Flumazenil infusion at a rate of 0.25 mg per hour has also been used to maintain arousal in a patient with severe benzodiazepine overdose, successfully tapered off after 28 days [4]. Flumazenil infusion, however, has not been shown to reduce the rate of complications such as airway compromise, pneumonia, and death. Seizures, nausea, and vomiting are some known side-effects of flumazenil [4], however, the use of flumazenil continuous infusion has generally been well-tolerated without a significant incidence of serious side effects [4,7]. Maintaining alertness in such patients may require prolonged taper to prevent a relapse into a less arousable state [4]. Flumazenil was safely used in our patient without such side effects, and the patient was able to be successfully extubated with a good clinical outcome.

## Conclusions

Alcohol withdrawal syndrome is commonly encountered in the clinical setting. The first-line treatment option for alcohol withdrawal is benzodiazepines, often used with symptom-triggered therapy and rapid escalation of dosing. Overdose of benzodiazepines can result in reduced arousal and respiratory depression, which may require mechanical ventilation. The risk is increased when long-acting benzodiazepines are used in obese patients, particularly with liver or kidney dysfunction, due to impaired metabolism and elimination. Flumazenil continuous infusion can be used for the reversal of sedation from a benzodiazepine overdose in a dose-dependent manner, sometimes requiring a prolonged taper to reduce reverting to a decreased arousal state.
